# Massive gastrointestinal stromal tumor with exophytic growth mimicking pelvic leiomyosarcoma: a case report and review of the literature

**DOI:** 10.1097/RC9.0000000000000054

**Published:** 2026-01-09

**Authors:** Ahmad Al-Bitar, Mhd Ammar Zalzaleh, Hussein Al Helbawi, Maher Saifo

**Affiliations:** a Faculty of Medicine, Damascus University, Damascus, Syrian Arab Republic; b Department of Internal Medicine, Damascus University, Damascus, Syrian Arab Republic; c Department of Radiology, Damascus Hospital, Damascus, Syrian Arab Republic; d Department of Oncology, Damascus University, Damascus, Syrian Arab Republic

**Keywords:** case report, exophytic growth, gastrointestinal stromal tumor (GIST), leiomyosarcoma (LMS), pelvic mass

## Abstract

**Introduction:**

Gastrointestinal stromal tumors (GISTs) are mesenchymal neoplasms of the gastrointestinal (GI) tract. Large, exophytically growing GISTs from the small bowel can present as massive abdominopelvic masses, creating a diagnostic challenge by mimicking primary gynecologic malignancies such as uterine leiomyosarcoma.

**Case Presentation:**

A 55-year-old woman presented with a 20-cm pelvic mass, initially diagnosed on computed tomography as a uterine leiomyosarcoma. Exploratory laparotomy, however, revealed the mass originated from the small bowel. Following resection, histopathology confirmed a malignant GIST with significant mitotic activity. The diagnosis was secured by strong immunohistochemical positivity for CD117 and DOG1. The patient remained disease-free at her 6-month follow-up.

**Clinical Discussion:**

This case highlights a critical diagnostic pitfall where a GIST’s exophytic growth pattern obscures its true origin on imaging. A high index of suspicion for GIST must be maintained in the differential for any large abdominopelvic mass, regardless of its apparent source. An accurate diagnosis requires integrating surgical findings with immunohistochemical profiling, which is paramount for guiding appropriate oncologic management.

**Conclusion:**

Massive small bowel GISTs can radiologically mimic primary pelvic malignancies. This case emphasizes including GIST in the differential diagnosis of large pelvic masses, even when imaging suggests a gynecologic origin. Definitive diagnosis relies on a multidisciplinary approach to ensure accurate classification and appropriate oncologic therapy.

## Introduction

Gastrointestinal stromal tumors (GISTs) are the most common mesenchymal neoplasms of the gastrointestinal (GI) tract^[1]^. A key biological feature is their tendency for exophytic growth, often originating from the muscularis propria layer and expanding outwards into the abdominal cavity^[2]^. When a small bowel GIST grows to a massive size, this pattern can obscure its intestinal origin on preoperative imaging^[3]^. Consequently, a large exophytic GIST can mimic a primary mesenteric, omental, or retroperitoneal mass, creating a radiological picture that is often mistaken for other sarcomas or, in the pelvis, gynecologic malignancies such as uterine leiomyosarcoma (LMS)^[4,5]^.

**HIGHLIGHTS**

- Large, exophytically growing small bowel GISTs can present as primary pelvic masses, closely mimicking gynecologic malignancies like uterine sarcoma.
- A high index of suspicion for GIST should be maintained for any large abdominopelvic mass, even when preoperative imaging suggests a gynecologic origin.
- Definitive diagnosis requires a multidisciplinary approach combining surgical exploration with specific immunohistochemical profiling (e.g., CD117, DOG1), which is crucial for guiding correct oncologic management.

This diagnostic distinction is critical, as the systemic therapies for GIST and LMS are fundamentally different. The vast majority of GISTs are driven by oncogenic mutations in the KIT or PDGFRA genes, making them highly sensitive to targeted tyrosine kinase inhibitors like imatinib^[6]^. In contrast, LMS, a true smooth muscle tumor, is resistant to this therapy and is managed with conventional chemotherapy. Therefore, accurate diagnosis is paramount for guiding appropriate oncologic management and has significant prognostic implications, as adjuvant imatinib can dramatically reduce recurrence risk in high-risk GIST^[7,8]^.

Herein, we present the case of a 55-year-old woman with a massive GIST whose exophytic growth into the pelvis led to a preoperative diagnosis of uterine LMS. This case report has been reported in line with the SCARE checklist^[9]^.

## Case presentation

A 55-year-old woman from the Middle East presented to our department with a 1-year history of progressively worsening abdominal distension and pressure symptoms, including a sensation of bladder fullness and increased urinary frequency. She reported no constitutional symptoms like weight loss or fever, nor any specific GI complaints such as bleeding or altered bowel habits. Of gynecological relevance, the patient was postmenopausal and reported no history of vaginal bleeding or discharge. Her past medical and surgical history was unremarkable. She was on no regular medications, had no known drug allergies, and her family history was non-contributory for malignancy. Socially, she was a non-smoker and did not consume alcohol.

**Figure 1.**
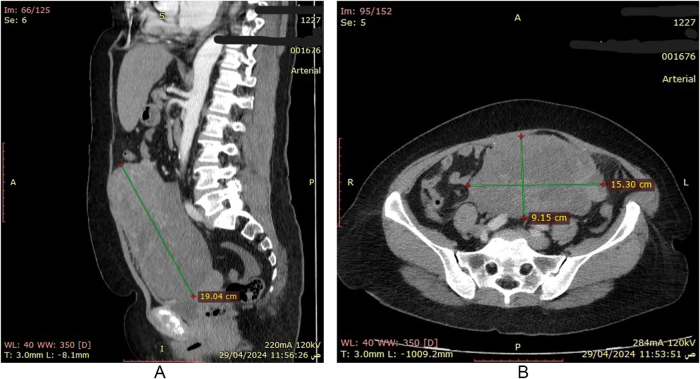
(A), (B) Axial and coronal views of contrast-enhanced CT scan before surgery showing a large, heterogeneous pelvic mass with central necrosis (arrows), initially suspected to be of uterine origin.

The diagnostic workup began with an abdominal ultrasound that identified a large pelvic mass, initially suspected to be a giant uterine fibroid. This was followed by a contrast-enhanced computed tomography (CT) scan, which revealed a massive 19-cm abdominopelvic mass with heterogeneous enhancement, a hypervascular peripheral rim, and extensive central necrosis (Fig. 1). The mass was contiguous with the uterus but showed no clear plane of origin from the bowel. The primary radiological differential diagnoses included uterine LMS, a degenerating uterine fibroid, ovarian carcinoma, and a GIST. A GIST remained a key consideration, given the classic exophytic growth pattern, heterogeneous enhancement with necrosis, and the absence of significant lymphadenopathy. Given the mass’s size, malignant radiological features, and the potential risks of a pre-operative biopsy (including hemorrhage and tumor seeding), a multidisciplinary team decision was made to proceed with diagnostic and therapeutic exploratory laparotomy.

An exploratory laparotomy was performed via a midline incision under general anesthesia by an experienced hepatobiliary surgical team. Intraoperative findings were pivotal: the massive tumor was adherent to the uterine serosa but was definitively originating from the antimesenteric border of the mid-jejunum. The initial surgical plan was therefore altered from a likely gynecologic resection to an en bloc resection of the tumor, including a segment of the involved small bowel and the adherent uterine serosa. A primary stapled anastomosis was created. The patient had an uncomplicated recovery with an estimated blood loss of 400 mL and was discharged on postoperative day 5 following an enhanced recovery protocol, with no postoperative complications (Clavien–Dindo Grade 0).

Histopathological analysis of the 1120-gram specimen confirmed a spindle cell GIST with a high mitotic rate of 15 mitoses/50 HPF. Immunohistochemistry was definitive, showing strong positivity for CD117 (c-KIT) and DOG-1. Based on the tumor size (>10 cm) and high mitotic rate, it was classified as a high-risk GIST according to the modified NIH criteria. Given this high-risk stratification, the patient was counseled and subsequently started on adjuvant imatinib therapy (400 mg daily), with a planned duration of 3 years.

**Figure 2.**
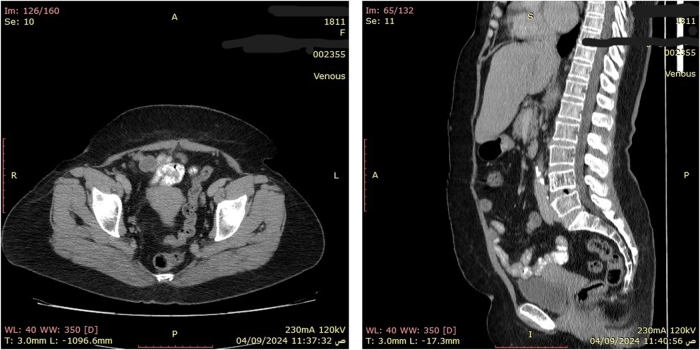
(A), (B) Follow-up contrast-enhanced CT scans at 6 months post-operatively showing no evidence of local recurrence or metastatic disease.

Her follow-up regimen includes clinical review and CT imaging every 3–4 months for the first 3 years (Figure 2). The first follow-up CT scan at 6 months post-surgery showed no evidence of local recurrence or metastatic disease, and she remains clinically well and adherent to her adjuvant therapy at her most recent 12-month visit.

## Discussion

This case exemplifies the profound diagnostic difficulty that can arise when a small bowel GIST exhibits massive exophytic growth into the pelvis, creating a clinical and radiological scenario that strongly mimics a primary gynecologic malignancy. The initial misinterpretation of the tumor as a uterine LMS underscores the limitations of preoperative imaging in specific anatomical contexts and highlights the indispensable role of surgical exploration and definitive histopathological analysis.

## The exophytic growth pattern and radiological hallmarks

Unlike epithelial cancers that grow into the lumen and cause early obstructive symptoms, GISTs arise from the muscularis propria and frequently grow outwards, away from the bowel lumen, in an “exophytic” or “exoenteric” fashion^[10]^. This pattern is especially common in the small bowel. As a result, these tumors can expand into the potential space of the abdominal cavity, reaching enormous dimensions – sometimes exceeding 30 cm – while causing minimal or no bowel obstruction. This silent, outward growth is the primary reason why a GIST can evolve from a small intestinal tumor into a massive, non-specific abdominopelvic mass, setting the stage for a profound diagnostic dilemma.

CT is the imaging modality of choice for diagnosing and staging GISTs. The radiological appearance of GISTs varies significantly with tumor size. Small GISTs (<5 cm) typically appear as sharply marginated, homogeneous, and hypervascular soft-tissue masses arising from the bowel wall^[11–13]^. However, large GISTs (>6 cm), such as the one in this case, present with more complex and characteristic features, as summarized in Table 1. They are typically well-defined, lobulated masses that demonstrate a predominantly extraluminal growth pattern. On contrast-enhanced CT, heterogeneous enhancement is the hallmark, reflecting a combination of viable tumor tissue and internal degeneration. This manifests as peripheral enhancement with large, central, non-enhancing areas of low attenuation corresponding to necrosis, hemorrhage, or cystic change. Mucosal ulceration occurs in up to 50% of cases and may lead to a fistulous communication, visualized as entrapped air or oral contrast within the mass^[14–19]^. Two negative findings are particularly valuable: the absence of significant lymphadenopathy and the uncommon presence of calcification.

**Table 1:** Characteristic CT features of large small bowel GISTs

CT feature	Description
Size	Often large at presentation, frequently >10 cm.
Growth pattern	Predominantly exophytic (exoenteric), growing away from the lumen. Bowel obstruction is rare despite a large size.
Enhancement	Heterogeneous, with avid peripheral enhancement and central low attenuation on contrast-enhanced scans.
Internal components	Central necrosis, hemorrhage, or cystic degeneration is common. Mucosal ulceration can lead to internal air or oral contrast.
Key negative findings	Absence of significant mesenteric or retroperitoneal lymphadenopathy. Calcification is uncommon.

The tumor in our patient exhibited many of these classic radiological signs. The diagnostic error in this case did not stem from atypical tumor features but rather from the confounding anatomical context. The tumor’s massive size and deep pelvic location obscured its pedicle of origin from the small bowel and created a broad interface with the uterus, leading to the logical but incorrect conclusion of a primary gynecologic sarcoma^[20,21]^.

## The diagnostic pitfall and the GIST vs extragastrointestinal stromal tumor dilemma

The literature contains numerous case reports documenting large GISTs that were preoperatively misdiagnosed as primary pelvic tumors^[16–19]^. In these cases, a massive exophytic GIST arising from a mobile loop of the small bowel descends into the pelvis. Its sheer bulk creates a broad interface with the uterus or adnexa, completely obscuring its narrow pedicle of origin. This anatomical deception leads to logical but incorrect preoperative diagnoses of uterine LMS, ovarian carcinoma, or a degenerating fibroid.

This diagnostic confusion is related to the concept of the extragastrointestinal stromal tumor (EGIST). An EGIST is defined as a tumor that is histologically and immunophenotypically identical to a GIST but arises primarily in the omentum, mesentery, or retroperitoneum with no connection to the GI tract wall^[22]^. However, the existence of “true” EGISTs is debated. A prevailing hypothesis suggests that many, if not most, tumors classified as EGISTs are actually exophytic GISTs that have detached from the gut wall or whose connection was too tenuous to be identified^[23,24]^. This highlights that a mass appearing to be “extragastrointestinal” on imaging should still carry a high suspicion for a GIST of GI origin. The key distinctions are summarized in Table 2.

**Table 2:** Comparison of GIST and EGIST

Feature	GIST (Gastrointestinal Stromal Tumor)	EGIST (Extragastrointestinal Stromal Tumor)
Definition	Arises from the wall of the GI tract.	Arises in the omentum, mesentery, or retroperitoneum with no connection to the GI tract wall.
Common locations	Stomach, small intestine.	Omentum, mesentery, retroperitoneum.
Origin hypothesis	Arises from interstitial cells of Cajal within the GI wall.	May be exophytic GISTs that detached from the gut wall.
Diagnostic challenge	Preoperative imaging can be misleading when exophytic growth obscures the origin.	Preoperative imaging often cannot distinguish between a primary pelvic sarcoma and an exophytic GIST; definitive origin is confirmed surgically.

## Definitive diagnosis: Histopathology and immunohistochemistry

While imaging raises suspicion, the definitive diagnosis of GIST and its differentiation from mimics like LMS rests on histopathology and immunohistochemistry. Morphologically, both GIST and LMS are spindle cell neoplasms, but subtle differences exist. GISTs typically consist of uniform spindle cells with pale, fibrillary cytoplasm, whereas LMS is characterized by cells with densely eosinophilic cytoplasm arranged in intersecting fascicles, often with more pronounced nuclear pleomorphism.

Immunohistochemistry, however, is the ultimate arbiter. Strong and diffuse positivity for CD117 (c-KIT), along with positivity for DOG1, is now considered pathognomonic for GIST. In contrast, LMS is characteristically negative for both CD117 and DOG1 but stains positively for smooth muscle markers such as desmin and smooth muscle actin (SMA). The key differentiating features are summarized in Table 3^[25–27]^.

**Table 3:** Differentiating features of small bowel GIST and pelvic leiomyosarcoma

Feature	Small bowel GIST	Pelvic leiomyosarcoma (LMS)
Cell of Origin	Interstitial cells of Cajal (or precursor)	Smooth muscle cell
Common Primary Site	GI tract (stomach, small bowel)	Uterus, retroperitoneum
Typical Growth Pattern	Exophytic, well-circumscribed	Often more infiltrative
Histologic Morphology	Spindle cells, pale/fibrillary cytoplasm, uniform nuclear atypia	Spindle cells, densely eosinophilic cytoplasm, fascicular pattern, marked pleomorphism
Key Positive IHC Markers	CD117 (c-KIT), DOG1	Desmin, smooth muscle actin
Key Negative IHC Markers	Desmin (usually negative)	CD117, DOG1

In our case, the strong positivity for CD117 and DOG1, combined with negativity for desmin, unequivocally confirmed the diagnosis of GIST and excluded LMS.

## Management and prognosis

The cornerstone of curative-intent therapy for localized GIST is complete surgical resection with negative margins (R0 resection). Given the tumor’s large size (>10 cm) and high mitotic activity, this patient’s GIST was classified as high risk for recurrence. In such cases, adjuvant therapy with a tyrosine kinase inhibitor, most commonly imatinib, for at least 3 years is the standard of care to reduce the risk of relapse^[28,29]^. The patient’s favorable outcome at one year is encouraging, but long-term oncologic surveillance with interval imaging is essential to monitor for potential recurrence, which most commonly occurs in the liver or peritoneum.

## Conclusion

This case highlights a critical diagnostic pitfall: a massive, exophytic small bowel GIST masquerading as a primary pelvic sarcoma. Its growth pattern can obscure the organ of origin, leading to misdiagnosis. A high index of suspicion for GIST is essential for any large abdominopelvic mass, particularly in the absence of significant lymphadenopathy. Definitive diagnosis requires a multidisciplinary approach integrating surgical exploration with histopathological and immunohistochemical analysis. This accurate classification is paramount, as it directly guides the appropriate oncologic management – specifically, the use of targeted tyrosine kinase inhibitors for GIST versus conventional chemotherapy for other sarcomas – to ensure optimal patient outcomes.
